# Identification and characterization of common B cell epitope in bovine leukemia virus via high-throughput peptide screening system in infected cattle

**DOI:** 10.1186/s12977-015-0233-x

**Published:** 2015-12-30

**Authors:** Lanlan Bai, Hiroyuki Otsuki, Hirotaka Sato, Junko Kohara, Emiko Isogai, Shin-nosuke Takeshima, Yoko Aida

**Affiliations:** Viral Infectious Diseases Unit, RIKEN, 2-1 Hirosawa, Wako, Saitama, 351-0198 Japan; Laboratory of Animal Microbiology, Department of Microbial Biotechnology, Graduate School of Agricultural Science, Tohoku University, Sendai, Miyagi 981-8555 Japan; Bovine Leukemia Virus Vaccine Laboratory, RIKEN Innovation Center, RIKEN, Wako, Saitama, 351-0198 Japan; Animal Research Center, Hokkaido Research Organization, Shintoku, Hokkaido 081-0038 Japan

**Keywords:** Bovine leukemia virus, Common B cell epitope, Comprehensive screening, Peptide microarray, Peptide ELISA high-throughput system, Antibody binding site, Bovine leukocyte antigen class II

## Abstract

**Background:**

Bovine leukemia virus (BLV) is the causative agent of enzootic bovine leukosis, the most common neoplastic disease of cattle. BLV is closely related to human T cell leukemia virus. B cell epitopes are important for the use of antibodies as therapeutic agents, the epitope-driven vaccine design, and immunological assays. A common B cell epitope for BLV has not yet been found due to individual differences in disease susceptibility.

**Results:**

We used a peptide microarray with 156 synthetic 15-mer peptides covering the envelope glycoprotein gp51 and the Gag proteins p15, p24, and p12 to map B cell epitope and one B cell epitope, gp51p16, was recognized by all four cattle experimentally infected with BLV. A newly developed high-throughput peptide ELISA system revealed 590 (91.2 %) of 647 cattle naturally infected with BLV, carrying 25 different bovine leukocyte antigen class II *DRB3* (*BoLA*-*DRB3)* alleles, responded to a 20-mer gp51p16-C peptide containing a C-terminal cysteine and gp51p16. Alanine mutation and comparison of the sequences at 17 amino acid positions within gp51p16-C revealed that R7, R9, F10, V16, and Y18 were the common binding sites to BLV antibodies, and two of these sites were found to be highly conserved. Transient expression in the cells of five infectious molecular clones of BLV with a single alanine mutation at five common antibody binding sites had no effect syncytia formation of the gp51 protein. In addition, the mutant proteins, R7A and R9A had no effect on the expression of gp51 protein; the gp51 protein expressions of F10A, V16A and Y18A were lower than that of the wild type protein.

**Conclusions:**

This is the first report to identify a common B cell epitope in BLV by comprehensive screening of BLV-infected cattle with varied genetic backgrounds in *BoLA*-*DRB3*. Our results have important implications for disease control and diagnosis.

## Background

Bovine leukemia virus (BLV) is the etiologic agent of enzootic bovine leukosis (EBL), the most common neoplastic disease of cattle, and is closely related to human T cell leukemia virus type 1 and 2 (HTLV-1 and HTLV-2) [1–3]. BLV is highly prevalent in several regions of the world and induces major economic losses in cattle production and export [4]. Erskine and Bartlett et al. had reported BLV infection is associated with herd-level in high-performing dairy herds and cow longevity [5, 6]. In 1995, losses in the dairy industry due to BLV from USA were estimated to be $US 525 million annually [4]. Recently study had been reported the economic losse per case of lymphosarcoma was estimated to be $412 [7].

BLV expresses the structural *gag*, *pol*, and *env* genes that are required for viral particle synthesis [8–10]. The *gag* gene of BLV is translated as the precursor Pr45 Gag and is processed into three mature proteins [8, 11]: the matrix protein p15 that binds genomic viral RNA and interacts with the lipid bilayer of the viral membrane [12]; the capsid protein p24 that is the major target for the host immune response with high antibody titers found in the serum of infected animals [13, 14]; and the nucleocapsid protein p12 that binds to the packaged genomic RNA [15]. The *env* gene encodes a mature surface glycoprotein (gp51) and a transmembrane protein (gp30) [8]. The N-terminal half of mature BLV gp51 plays an important role in viral infectivity and syncytium formation [16–18].

Virus factors and host factors are believed to determine disease progression in chronic infectious diseases such as acquired immunodeficiency syndrome (AIDS), HTLV-associated diseases, and BLV-induced EBL [19]. One of the most important host factors is the polymorphism of the major histocompatibility complex (MHC). In cattle, the MHC system is known as the bovine leukocyte antigen (BoLA) system and is a highly polymorphic and tightly linked gene cluster [20]. There is one predominant class II *DRB* locus in cattle, namely, *BoLA*-*DRB3* [21], and this locus is the most polymorphic class II locus in cattle. To date, 132 alleles have been registered on the Immuno Polymorphism Database (IPD)-MHC database (http://www.ebi.ac.uk/ipd/mhc/bola). It has been suggested that the *BoLA*-*DRB3* gene may play a direct role in controlling the number of BLV-infected peripheral B lymphocytes in vivo in Holstein cattle [22, 23]. Moreover, the *BoLA*-DRB3*0902 and *BoLA*-DRB3*1101 polymorphisms were reported to be associated with a low proviral load (LPVL), and *BoLA*-DRB3*1601 was associated with a high proviral load (HPVL) in Japanese Black cattle [24]. Thus, individual differences in cattle against BLV disease progression are believed to be determined by highly polymorphic BoLA class II alleles.

By epitope mapping using cattle with experimentally infected BLV and a library of overlapping peptides of BLV, we identified CD8^+^ cytotoxic T lymphocyte epitopes [25] and CD4^+^ T cell epitopes (Unpublished Data) that were found to differ from those previously identified in cattle, mice, and sheep [17, 26–29]. Therefore, we hypothesized that not only animal species but also individual differences affect BLV epitopes. T cell epitopes of BLV have been identified in previous studies [17, 26–29], and these epitopes have been not found as a common epitope for all BLV-infected cattle. The large individual differences in BLV immunoreactivity pose a major challenge in the development of a vaccine against BLV.

Previous studies have reported that humoral immunity is induced in earlier phases of infection in BLV-infected animals [30–33]. The B cells of the immune system recognize the pathogen’s antigens by their membrane-bound immunoglobulin receptors and, in response, produce antibodies specific to these antigens; antibodies bind to antigens at specific sites that correspond to the antigenic determinants or B cell epitopes [34–36]. Therefore, identification and characterization of common B cell epitopes in target antigens is a key step in antibody production, epitope-driven vaccine design, and immunodiagnostic tests. In the present study, identification of a common B cell epitope in BLV among a large population of BLV-infected cattle with varied genetic backgrounds in *BoLA*-*DRB3* alleles was investigated. First, 156 synthetic peptides that covered the entire Gag protein and gp51 protein sequence were prepared for the identification of common B cell epitopes in BLV that respond to all four cattle experimentally infected with BLV via a peptide microarray that was highly specific to the antigen–antibody response. Second, a new high-throughput peptide enzyme-linked immunosorbent assay (ELISA) system was established for confirmation of whether the identified B cell epitope was common via analysis of serum samples from cattle carrying 25 different *BoLA*-*DRB3* alleles. Third, the possible common binding sites to BLV antibodies were determined using variants harboring a single alanine mutation on each amino acid position within the common B cell epitope. Fourth, the effects of alanine substitution of a common binding site within the common B cell epitope on viral expression and fusogenicity were examined by reverse genetics of an infectious molecular clone of BLV, pBLV-IF [37, 38]. Finally, the conservation of each amino acid within the common B cell epitope was examined.

## Results

### Identification of a common B cell epitope via a high-throughput peptide ELISA system

To identify a B cell epitope in BLV, 156 synthetic peptides of 15 amino acids in length that were derived from the whole BLV Gag proteins, p12, p24, and p15, and the Env gp51 protein, were prepared, followed by peptide microarray [39]. Using peptide microarray, the responses between the negative and positive serum samples obtained before and after experimental infection of four cattle, JBS4, JBS6, JBN1, and JBN2, that carried the *BoLA*-DRB3*1601, *1501, *2703, or *0503 alleles (Table 1), resulted in the detection of B cell epitopes that responded with the positive serum from each cattle (Table 3). Only one B cell epitope was recognized by all of the experimentally infected cattle and was termed gp51p16, reasons for individual difference in disease susceptibility against BLV that may be associated with polymorphisms of the *BoLA*-*DRB3* gene.

**Table 1:** Japanese black cattle examined in peptide microarray

Animal no.^a^	BoLA-DRB3^b^ alleles A; B	Age (month)	Sex	WBCs^c^ (cells/μl)		PBLs^d^ (cells/μl)		Proviral load^e^ (copies per 10^5^ cells)		Antibodies to BLV^f^	
				BLV-challenged		BLV-challenged		BLV-challenged		BLV-challenged	
				Before	After	Before	After	Before	After	Before	After
JBS4	1601; 1601	8	♂	10,200	15,500	6560	11,100	0	67,284	−	+
JBS6	1601; 1601	6	♂	9170	13,900	4690	9570	0	93,456	−	+
JBN1	1501; 2703	6	♀	9060	8250	3084	4900	0	149	−	+
JBN2	1501; 0503	6	♀	11,800	12,100	8142	9140	0	58,176	−	+

*−* BLV negative serum; *+* BLV positive serum

^a^Four BLV-negative Japanese Black calves (6–8-months-old) were experimentally challenged intravenously with white blood cells containing a proviral load of 4 × 10^7^ proviral copies, as determined by CoCoMo-qPCR-2 assay

^b^ *BoLA*-*DRB3* alleles were genotyped by PCR-SBT method and are according to the BoLA nomenclature committee of the Immune Polymorphism Database (IPD)-MHC database. Both alleles, A and B, for each experimental animal was shown

^c^White blood cells (WBCs) were measured at a single time point in all calves during the experiment

^d^Peripheral blood lymphocytes (PBLs) were measured at a single time point in all calves during the experiment

^e^Proviral load (expressed as the number of copies of provirus per 10^5^ peripheral blood mononuclear cells) was evaluated using CoCoMo-qPCR-2 assay

^f^ELISA (enzyme-linked immunosorbent assay) to detect anti-BLV antibodies was performed using the BLV ELISA kit

**Fig. 1:**
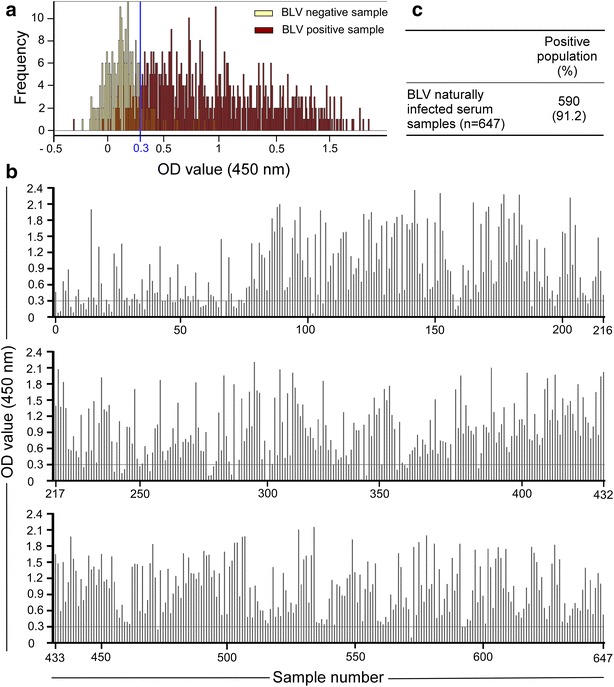
Results of the analyses of gp51p16-C with BLV positive serum. These experiments were performed by a new high-throughput peptide ELISA system. **a** The define line of positive response. The *line* represents a positive response with an OD value of 0.3 that was determined in accordance to histogram analysis using 270 BLV negative and 647 BLV positive serums as described above Table 2. The average and standard deviation of negative samples were 0.12 and 0.16, and postive samples were 0.96 and 0.54, respectively. **b** The response of gp51p16-C with positive serum from 647 cattle naturally infected with BLV by ELISA. Serum samples collected from cattle naturally infected with BLV were observed to have different levels of proviral loads and shared 25 different *BoLA*-*DRB3* alleles. **c** Positive population of 647 serum samples from BLV naturally infected cattle with gp51p16-C

To clarify whether gp51p16 is a common B cell epitope, a new high-throughput peptide ELISA system utilizing maleimide-activated carrier protein mcKLH linked with the thioether bond of the terminal cysteine of gp51p16-C that was modified as a C-terminal cysteine-containing 20-mer peptide to fix gp51p16 on a mcKLH-coated microplate was developed. Comprehensive screening of serum samples obtained from 647 cattle naturally infected with BLV that shared 25 different *BoLA*-*DRB3* alleles and 270 BLV non-infected cattle that shared 23 different *BoLA*-*DRB3* alleles were performed (Table 2). The 20 alleles were same among the non-infected group and infected group. We have defined the line of positive response by histogram analysis using 917 serum samples (Fig. 1a). The frequency of negative samples mostly declined and crossed with positive samples’ was as determination point, and that OD value was 0.3. In 590 (91.2 %) of 647 serum samples, gp51p16-C was found to be positive in cattle that were positive for anti-BLV antibody using an anti-BLV ELISA kit and BLV provirus using BLV-CoCoMo-qPCR-2 (Fig. 1). These results suggest that gp51p16-C contains a common B cell epitope in a large number of cattle naturally infected with BLV carrying 25 different *BoLA*-*DRB3* alleles.

**Table 2:** The serums used in a new peptide ELISA high-throughput system

Local	Farm no.	BLV positive samples^a^			BLV negative samples^b^		
		Cattle no.	*BoLA*-*DRB3*		Cattle no.	*BoLA*-*DRB3*	
			No.	Alleles		No.	Alleles
Aichi	2	32	12	0101, 0201, 0502, 0503, 0701, 0902, 1001, 1101, 14011, 1501, 1601, 1801	5	6	0201, 0503, 1101, 1302, 1501, 1601
Ishikawa	1	12	7	0101, 0201, 0902, 1001, 1101, 1201, 1501	3	5	0601, 1101, 1201, 1501, 2703
Iwate	2	47	14	0101, 0201, 0502, 0503, 0701, 0902, 1001, 1101, 1201, 14,011, 1501, 1601, 2703, 4401	14	7	0503, 1001, 1101, 1201, 14011, 1501, 1601
Oita	2	30	13	0101, 0201, 0301, 0503, 0701, 0801, 0902, 1001, 1101, 1302, 1501, 1601, 2703	8	7	0101, 0201, 0301, 1001, 1101, 1201, 2703
Osaka	2	52	12	0101, 0201, 0701, 0902, 1001, 1101, 1201, 14011, 1501, 1601, 1801, 2703	2	4	0101, 1101, 14011, 1501
Okinawa	2	43	12	0101, 0201, 0502, 0503, 0701, 0902, 1001, 1101, 1201, 14011, 1501, 1601	19	10	0101, 0201, 0503. 0701, 1001, 1101, 1201, 14011, 1501, 1601
Kagoshima	2	9	7	0502, 0503, 0701, 1001, 1302, 1501, 1601	28	10	0101, 0502, 0503, 0801, 1001, 1101, 1201, 1302, 14011, 1501, 1601, 2703
Kanagawa	2	55	11	0101, 0201, 0701, 0902, 1001, 1101, 1201, 14011, 1501, 1801, 2703	10	8	0101, 0902, 1001, 1101, 1201, 14011, 1501, 2703
Gifu	3	67	17	0101, 0201, 0501, 0502, 0503, 0701, 0801, 0902, 1001, 1101, 1201, 1302, 14011, 1501, 1601, 1801, 2703	1	2	0101, 1001
Nagano	2	14	12	0101, 0501, 0502, 0901, 0902, 1001, 1101, 1201, 14011, 1501, 1601, 1801	7	2	0101, 0902, 1001, 1101, 14011, 1601, 2703
Shiga	2	24	10	0101, 0601, 0902, 1001, 1101, 1201, 14011, 1501, 1801, 2703	5	6	0101, 1001, 1101, 1201, 14011, 2703
Shizuoka	3	47	12	0101, 0201, 0503, 0901, 0902, 1001, 1101, 1201, 14011, 1501, 1601, 2703	13	10	0101, 0502, 0503, 0701, 0902, 1001, 1101, 1201, 1501, 1601
Hyogo	2	37	11	0101, 0201, 0902, 1001, 1101, 1201, 14011, 1501, 1601, 1801, 2703	30	10	0101, 0201, 0901, 0902, 1001, 1101, 1201, 14011, 1501, 1601, 1801, 2703
Chiba	2	45	13	0101, 0201, 0601, 0902, 1001, 1101, 1201, 14011, 1501, 1601, 1701, 1801, 2703	16	12	0101, 0601, 0701, 0902, 1001, 1101, 1103, 14011, 1501, 1701, 2703, 3201
Tokyo	1	16	6	0101, 1001, 1101, 14011, 1501, 1801	15	10	0101, 0902, 1001, 1101, 1201, 14011, 1501, 1601, 1801, 2703
Nagasaki	2	22	11	0101, 0503, 0601, 0701, 0902, 1001, 1101, 1201, 14011, 1501, 1601	43	16	0101, 0201, 0502, 0503, 0601, 0701, 0801, 0902, 1001, 1101. 1201, 14011, 1501, 1601, 2703, 3401
Niigata	2	42	15	0101, 0502, 0503, 0504, 0701, 0902, 1001, 1101, 1201, 14011, 1501, 1601, 2002, 2703, 3401	15	14	0101, 0502, 0503, 0701, 0902, 1001, 1101, 1103, 1201, 1302, 14011, 1501 1601, 2802
Hokkaido	1	9	9	0101, 0201, 0502, 0503, 1001, 1201, 14011, 1601, 3401	5	6	0502, 0503, 1001, 1201, 1501, 1601
Mie	1	3	4	0101, 1001, 1101, 1501	20	11	0101, 0201, 0503, 1001, 1101, 1201, 1302, 14011, 1501, 1601, 1701
Yamagata	2	41	10	0101, 0502, 0503, 0701, 1001, 1201, 14011, 1501, 1601, 2703	11	9	0201, 0502, 0701, 0801, 1001, 1101, 1201, 1501, 1601
Total	38	647			270		

Serum samples were obtained from 647 BLV naturally infected cattle which were positive for anti-BLV antibodies and BLV provirus as determined by an anti-BLV ELISA kit and a BLV-CoCoMo-qPCR-2 assay, respectively, and 270 BLV non-infected cattle

^a^These BLV positive samples had 25 different *BoLA*-*DRB3* alleles that were typed by PCR-SBT method

^b^These BLV negative samples had 23 different *BoLA*-*DRB3* alleles, which 20 alleles of them were same with identification alleles on BLV positive samples as described above (b)

### Binding site of the B cell epitope with BLV antibodies

**Fig. 2:**
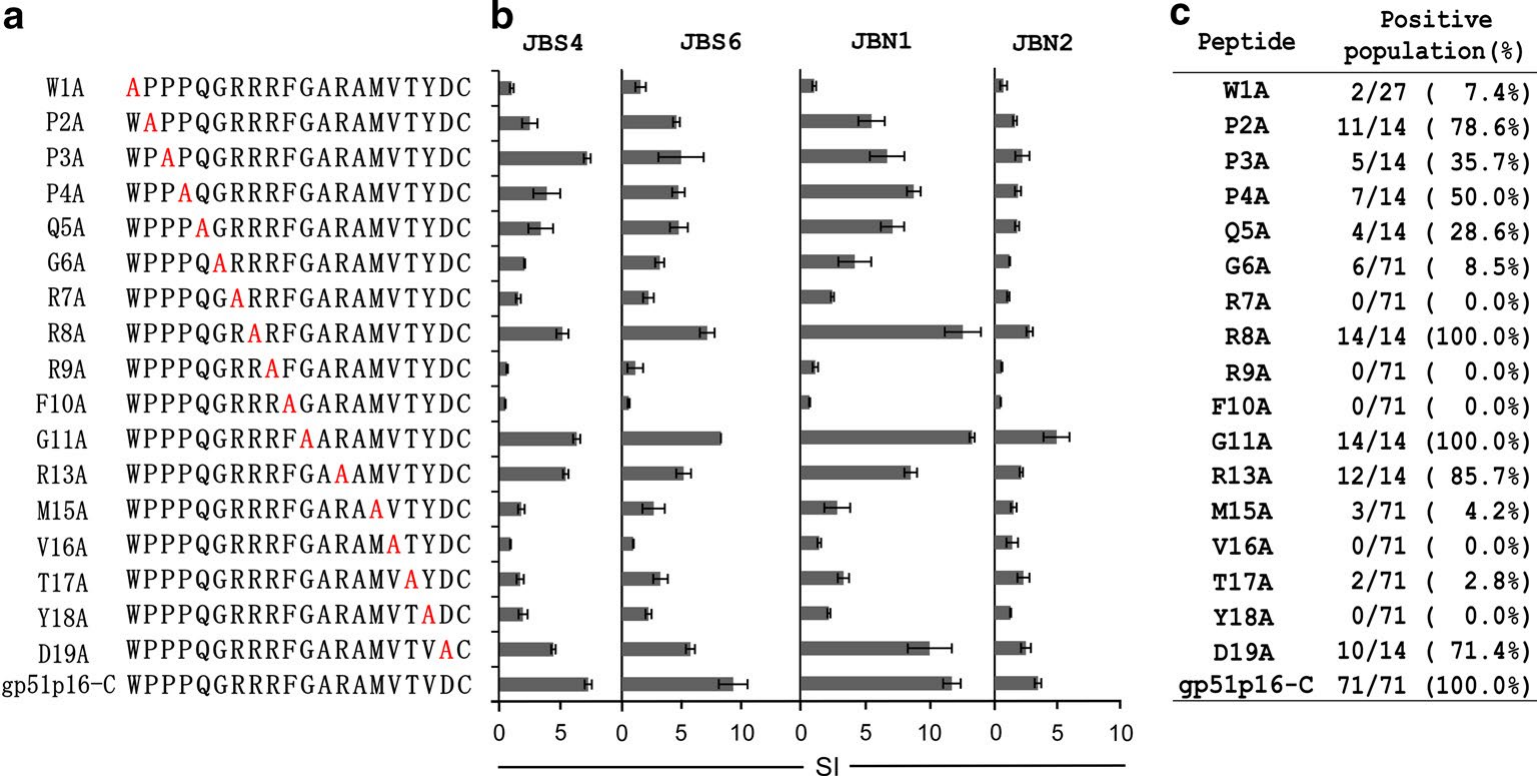
Response of BLV positive serum with single alanine variants of gp51p16-C. These experiments were performed by a new high-throughput peptide ELISA system. **a** Sequences of the gp51p16-C peptide and its variants with single alanine mutations are shown in the single-letter amino acid code, and alanine mutation sites are represented in *red*. **b** The stimulation index (SI) of four BLV positive serum samples was obtained from cattle experimentally infected with BLV (JBS4, JBS6, JBN1, and JBN2) for individual variants. **c** Reactions of the serum samples obtained from cattle naturally infected with BLV for individual variants

Antigen–antibody interactions play a pivotal role in the humoral immune response [35, 40]. To determine binding sites with BLV antibodies within the common epitope gp51p16-C, 17 variants harboring single alanine substitutions at each amino acid position within gp51p16-C were synthesized and named W1A to D19A, respectively (Fig. 2a). BLV positive serum was examined via a new high-throughput peptide ELISA system using these variants.

Figure 2b demonstrates the SI of BLV positive serum samples that were obtained from four cattle experimentally infected with BLV for each single alanine variant. The SI values of the P2A, P3A, P4A, Q5A, R8A, G11A, R13A, and D19A variants against the four BLV positive serum samples were observed higher than value of no peptide. By contrast, the W1A, G6A, R7A, R9A, F10A, M15A, V16A, T17A, and Y18A variants were found to have similar (same or less than) value of no peptide against the four BLV positive serum samples.

As shown in Fig. 2c, responses against four BLV positive serums of the 17 single alanine variants were further confirmed using a number of serum samples from cattle naturally infected with BLV (Table 2). The variants R7A, R9A, F10A, V16A, and Y18A were found to demonstrate similar (same or less than) value of no peptide in the 71 samples tested. These results suggest that the amino acid positions 7(R), 9(R), 10(F), 16(V), and 18(Y) within gp51p16-C may be common binding sites for anti-BLV antibodies.

### Effect of alanine substitutions within gp51p16-C in pBLV-IF on viral expression and syncytia formation

The five common binding sites for BLV antibodies within the common epitope gp51p16-C were determined using a peptide ELISA. Interestingly, the gp51p16-C epitope is located in the N-terminal half of the mature BLV gp51 protein that plays an important role in viral infectivity and syncytium formation [16–18]. To examine the effect of the alanine mutations corresponding to five common binding sites for BLV antibodies on Env protein expression and syncytium formation, the infectious molecular clone pBLV-IF was used [37]. To verify the amino acid sequences of gp51p16-C that are encoded by pBLV-IF, the nucleotide sequence of the gp51 gene of pBLV-IF that corresponded to gp51p16-C was determined and compared with the reported sequences of the BLV cell line FLK-BLV strain pBLV913 (GenBank accession no. EF600696). Comparison of the sequences revealed that the gp51 gene of pBLV-IF exhibited a high degree of identity in the nucleotide sequence (99 %) and the predicted amino acid sequence (100 %) to the gp51 protein in the pBLV913 strain [41].

**Fig. 3:**
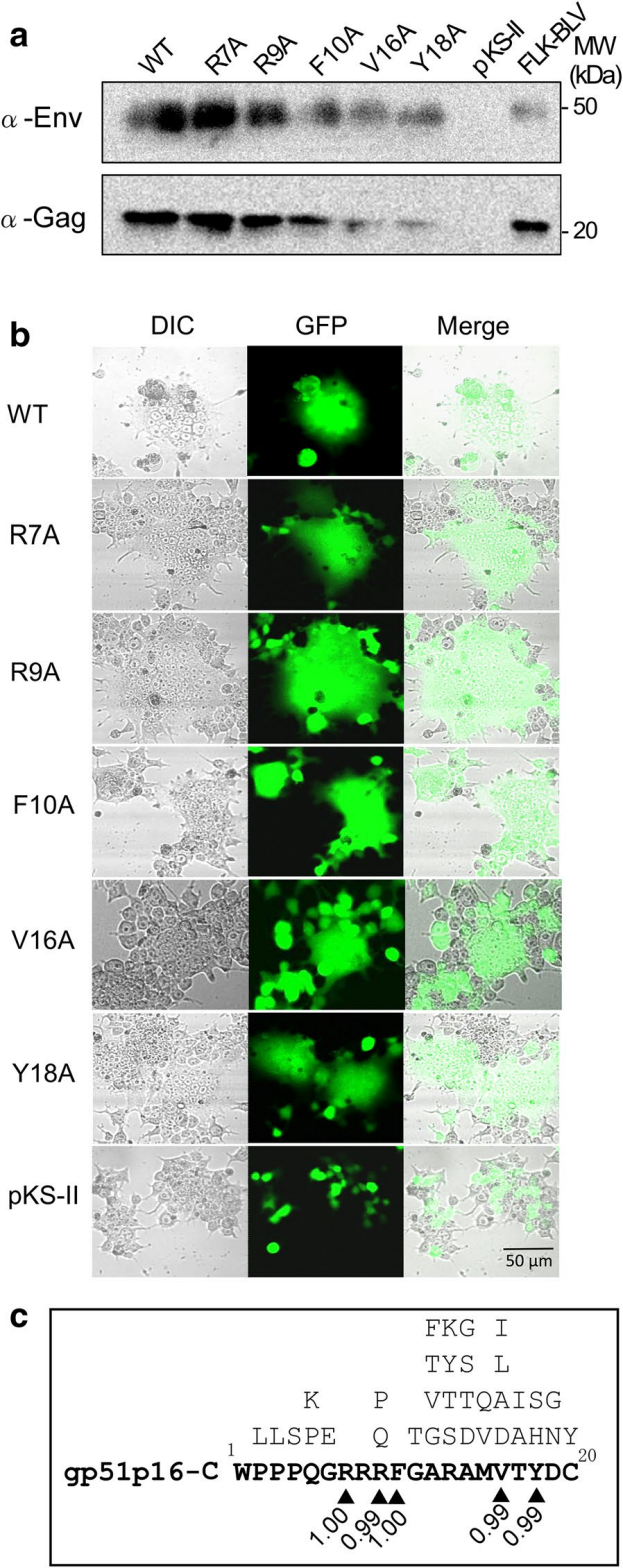
Effect of alanine substitutions in gp51p16-C of pBLV-IF on viral expression and fusogenicity, and conservation. After transfection, pBLV-IF, mutated pBLV-IFs with the single alanine mutations (R7A, R9A, F10A, V16A, and Y18A), or a negative control vector and the GFP expression vector pEGFP-N1 were performed Western blotting (**a**) and syncytium (**b**) analyses. GFP was used as the reporter molecule for discrimination between transfected and untransfected cells. **a** For Western blotting, a fraction of 23CLN cells were harvested 20 h following transfection and were analyzed for the ratio of GFP-expressing cells by flow cytometry. The remaining 23CLN cells were lysed 48 h after transfection, and lysates with equal numbers of GFP-expressing cells were subjected to Western blotting using anti-BLV Env protein and anti-Gag protein monoclonal antibodies and HRP-conjugated goat antibodies. FLK-BLV cells, which were productively infected with BLV, were used as the positive control. Positions of protein marker and BLV glycoprotein (gp51) were indicated and BLV Gag protein (p24). **b** For syncytium formation, 293T cells were visualized by confocal laser scanning microscopy. **c** Distribution of polymorphic and frequencies of antibody binding amino acid residues in gp51p16-C. BLV nucleotide sequences (n = 517) were collected from GenBank, and amino acid sequences corresponding to gp51p16-C were aligned. The amino acid positions 7, 9, 10, 16, and 18 are indicated by triangle. A single letter above gp51p16-C sequences indicates a polymorphic amino acid residue

The codons at the positions 7, 9, 10, 16, and 18 within gp51p16-C were mutated to the codon for an alanine residue(s) to obtain mutant forms of pBLV-IF. To examine expression of cell-associated Env and Gag proteins by these various mutants, we transiently transfected 23CLN cells that strongly express BLV antigens after transfection with pBLV-IF [37] with either wild-type pBLV-IF, its variants, or a negative control vector together with GFP expression vector pEGFP-N1 for normalization of the efficiency of transfection. Twenty hours following transfection, a fraction of 23CLN cells were harvested and GFP-expressing cells were analyzed by flow cytometry. Thirty-six hours after transfection, the remaining 23CLN cells were lysed and lysates containing equal numbers of GFP-expressing cells were subjected to Western blotting with anti-BLV Env and Gag proteins Mabs (Fig. 3a). Bands corresponding to the Env protein, gp51, and Gag protein, p24, of cell-associated BLV were specifically detected in cells that had been transfected with the mutated pBLV-IFs. The molecular mass of these proteins was similar to that of proteins detected in a lysate of FLK-BLV cells that had been productively infected with BLV [42] as a positive control. In addition, two gp51 mutant proteins, namely, R7A and R9A, were expressed at levels similar to that of the wild type gp51 protein, while the levels of expression of the other three mutant proteins, namely F10A, V16A, and Y18A were less than that of the wild type protein. Likewise, the expression of Gag protein is the similar to that of gp51 expression (Fig. 3a).

We next investigated whether the mutant forms of pBLV-IF comprise a fusion capacity, one of the cytopathic effects of BLV Env in 293T cells, which are able to induce formation of multinucleated cells. pBLV-IF and the mutated pBLV-IFs, or a negative control vector, combined with the GFP expression vector pEGFP-N1, were expressed in 293T cells, and GFP positive cells were visualized by confocal laser scanning microscopy. GFP was used as the reporter molecule for discrimination between transfected and untransfected cells. The results of a typical experiment are depicted in Fig. 3b. A fraction of the GFP positive cells with the single alanine mutated pBLV-IFs, R7A, R9A, F10A, V16A, and Y18A, showed an ability to form syncytium in addition to transfectant with wild-type pBLV-IF. By contrast, GFP positive cells transfected with the control vector were not observed to form syncytium.

These results demonstrate that the R7A and R9A mutations at common binding sites to BLV antibodies did not affect the synthesis, processing, and expression of viral proteins, and fusogenicity following transfection. F10A, V16A, and Y18A decreased the expression of gp51 protein.

### Distribution of polymorphic and conserved amino acid residues in gp51p16-C

To investigate the conservation of each amino acid residue within gp51p16-C between the BLV strains, the nucleotide sequences of 517 BLV strains were collected from GenBank and analyzed for the conservation of each amino acid and frequencies of binding amino acid within the common epitope gp51p16-C (Fig. 3c). Two (positions 7 and 10) of five common binding sites to BLV antibodies within the common epitope gp51p16-C were found to be highly conserved as there were no mutations at the amino acid positions 7 and 10 in the 517 BLV strains. The remaining three (positions 9, 16, and 18) were found to be polymorphic and the frequency was 0.99.

## Discussion

In this study, from 156 synthetic peptides encompassing the Gag and Env gp51 proteins, one B cell epitope, gp51p16, was recognized by all four cattle experimentally infected with BLV using a peptide microarray. The sequence of gp51p16 was PPQGRRRFGARAMVT. Serums from 647 cattle naturally infected with BLV that shared 25 different *BoLA*-*DRB3* alleles were used to examine the positive response of 20-mer gp51p16-C that comprised the entire length of the original gp51p16 (15-mer) and cysteine of C-terminal. Interestingly, gp51p16-C responded to 590 (91.2 %) of 647 serum samples. The corresponding sequence was WPPPQGRRRFGARAMVTYDC, indicating that gp51p16-C contains a common B cell epitope. In a previous study [17], synthetic peptides corresponding to the BLV gp51 protein were used to analyze B cell neutralizing and T helper cell epitopes, and the following epitopes were identified: Peptide 64–73 (RRRFGARAMV), 98–117 (SQADQGSFYVNHQILFLHLK), and 177–192 (PDCAICWEPSPPWAPE). Using rabbit serum, these were determined to be B cell neutralizing epitopes and Env pep61 epitope (PQGRRRFGARAMVTYDCE) was identified as a sequence capable of inducing T cell proliferation or mitogenicity in mice [27, 28]. In particular, the common B cell epitope identified in this study contained the whole B cell neutralizing epitope 64–73 that had been previously found in rabbit [17] and the majority of the Env pep61 epitope that had been previously identified in mice [27, 28]. Although a striking feature of gp51p16-C was the response to over 91.2 % of serum samples from naturally infected cattle, 57 (8.8 %) of 647 serum samples showed very weak responses with gp51p16-C. Two helper T cell epitopes (169–188, LNQTARAFPDCAICWEPSPP; and 177–192, PDCAICWEPSPPWAPE) that had been previously identified in cattle and 177–192 (PDCAICWEPSPPWAPE) that had been recognized as a B cell neutralizing epitope in rabbit indicated that bovine T cell epitopes may commonly share identical sequences with B cell neutralizing epitopes [17]. We hypothesized that the common B cell epitope, gp51p16-C, produces BLV antibodies, and therefore, helper T cell immunity is primarily induced in vivo in animals that had no positive responses to gp51p16-C.

In our study, CD4^+^ T cell epitope mapping was performed in Japanese Black cattle experimentally infected with BLV that carried different *BoLA*-*DRB3* alleles, and CD4^+^ T cell epitopes (unpublished data) were identified that differed to those previously reported in mice, sheep, and cattle [17]. In addition, the recombinant vaccinia virus expressing Env vaccine had reduced the proviral load in vaccinated sheep used as an experimental model, nonetheless it had not effected in cows [43, 44]. These results suggest that in addition to animal species, individual differences in cattle affect the BLV epitope. One important factor that induces individual differences is the polymorphism of MHC. Notably, the capacity of MHC class II molecules to deliver signals that anti-MHC class II Mabs may mediate several activities that include replacing T helper cells in the activation of B cells, thereby inducing the secretion of interleukin-1 in monocytes [45]. Staphylococcal exotoxins provide a superantigenic ‘bridge’ between T cell receptor molecules on T cells and MHC class II molecules on B cells, leading to B cell proliferation and immunoglobulin production that has been thought to trigger MHC-unrestricted interaction between T cells and B cells, leading to the onset of autoimmunity [46]. Furthermore, the *BoLA* class II *DRB3* allele has been reported to be associated with BLV proviral load [24]. One of the major challenges of the present study was the use of a large sample size of cattle with varied genetic backgrounds in *BoLA*-*DRB3* for the successful identification of a common B cell epitope.

The common B cell epitope is an important target for antibody production and vaccine antigens. Antibodies directed against peptide 64–73 (RRRFGARAMV) that are identical to most of gp51p16-C corresponding to the HTLV-I gp46 sequence (peptide 88–98; WTKIKPNRNGGGJ) were previously demonstrated to possess a neutralizing character [17, 47]. Therefore, we determined the binding sites to BLV antibodies within gp51p16-C using single alanine variants and serum from 71 cattle naturally infected with BLV. The common binding sites of gp51p16-C R(7), R(9), F(10), V(16), and Y(18) were identified. Importantly, two regions (peptides 64–73 and 78–92) of the BLV Env protein are included in the small domain forming the junction between the stem and the large globular head [48]. Therefore, the five antibody binding sites identified within gp51p16-C were considered as amino acids essential for the expression of the BLV Env protein.

Epitope antibodies may mediate inhibition of BLV-induced syncytium formation [17]. Several variants of BLV Env proteins have been reported as failing to induce syncytia resulting in sustained substitution of an amino acid conserved at analogous positions in the HTLV-1 Env protein [49]. Moreover, the peptide 64–73 that was found to be completely identical to most of gp51p16-C, had been previously reported to be adjacent to residue 62, which was located in epitope F of BLV gp51, may be linked to syncytium formation induced by BLV Env [17], and antisera raised against peptide 64–73 compete with Mab F recognition in an ELISA [16]. Hence, we examined whether the antibody binding sites identified within the common B cell epitope affect Env expression and cell fusion by reverse genetics of the infectious molecular clone pBLV-IF. R7A and R9A mutations were not observed to affect Env protein expression and syncytium formation. Likewise, in recently report [50], mutation of a single Env N-linked glycosylation site have not decreased pathogenicity of BLV has been reported. By contrast, F10R, V16A and Y18A have a little reduced the expression of gp51 proteins.

The Env protein strain-specific substitution Y108H, corresponding to Y18 in the gp51p16-C determined in the present study, has been exposed on the front face of the BLV receptor binding domain (RBD) [49]. In this study, this site was found to be polymorphic. The remaining binding sites were predicted to be exposed on the front face of BLV RBD. Furthermore, the amino acid positions 7 (R) and 10 (F) of gp51p16-C are highly conserved, indicating that they may be essential for unknown viral functions.

It is well known that pathogens may easily escape host immune recognition and may result in species-specific responses in humans and animals. The results reported in this study are not only of fundamental biological interest, but also of practical importance. This is the first study to report the presence of a common B cell epitope in BLV gp51 that is recognized by cattle with overlapping *BoLA*-*DRB3* alleles. BLV gp51 was characterized by an amino acid mutation of several BLV antibody binding sites that R7A and R9A were not found to have an effect on gp51 protein expression and fusogenicity. The remaining three antibody binding sites, F10A, V16A, and Y18A related with the reduction of expression of gp51 protein, not yet affect the syncytium formation. Therefore, the common B cell epitope gp51p16-C is the optimal target for the production of Mab, antigen for BLV vaccine design, and immunodiagnostic tests.

## Conclusions

One common B cell epitope, gp51p16, was identified by epitope mapping for 20-mer overlapping peptides of p12, p24, p15 and gp51 proteins. Gp51p16-C, which contains whole sequence of gp51p16 and C-terminal cysteine, was recognized common B cell epitope that showed 91.2 % positive response in a comprehensive screening of 647 BLV naturally infected cattle carrying 25 different *BoLA*-*DRB3* alleles. Furthermore, it contains five common antibody binding sites (amino acids R7, R9, F10, V16, and Y18) and which two common (R7 and F10) antibody binding sites were highly conserved. R7A and R9A have no effect on the expression and all of five antibody binding sites have no effect fusogenicity of BLV gp51 protein. The B cells of immune system plays an important role in preventing and suppressing the progression of disease caused by BLV. Thus, common B cell epitope, gp51p16-C, is particularly important to provide a useful target for monoclonal antibody production, antigens for the diagnosis of BLV infection, BLV vaccine strategies, and becomes a significant implication for disease control and diagnosis.

## Methods

### Animals, serum, and DNA extraction

For peptide microarray, serum samples were obtained from four Japanese Black cattle experimentally infected with BLV prior to infection and 21 weeks following BLV infection. BLV negative and positive serum samples were also obtained (Table 1). Blood samples were collected for a period of 10 weeks following inoculation, and DNA was extracted to measure proviral loads using the BLV-CoCoMo-qPCR-2 assay as previously described [51–54] (Table 1). BLV infection was examined using an anti-BLV antibody ELISA kit (JNC Inc., Tokyo, Japan) (Table 1). This study was approved by the local Animal Ethics Committee and the Animal Care and Use Committee of the Animal Research Center, Hokkaido Research Organization (approval number 1302). Furthermore, 647 BLV positive serum samples and 270 BLV negative serum, which were collected from 38 farms in 21 provinces in Japan used for this study (Table 2).

### Identification of *BoLA*-*DRB3* by polymerase chain reaction-sequence-based typing (PCR-SBT)

*BoLA*-*DRB3* alleles were typed using the PCR-SBT method as previously described [55].

### B cell epitope mapping using peptide microarray

The PepStar peptide microarrays were provided by JPT Peptide Technologies (Berlin, Germany) [56]. A library was constructed of 156 overlapping peptides comprising 15-mer peptides with an offset of four amino acids that were derived from mature peptides encoded and expressed in *gag* and *env*, the BLV Gag proteins, p12, p24, and p15 corresponding to the reported sequences of BLV Gag (GenBank accession no. LC057268), and BLV Env gp51 corresponding to the reported sequences of Env (GenBank accession no. EF600696). The primary sequence of the amino acid positions (Table 3) was commercially synthesized using the PepStar technique. The synthesized peptides were displayed in three identical subarrays on each slide. The peptide microarray slide and glass cover were prepared for the microarray sandwich and blocked with Sea Block Blocking Buffer (Thermo Fisher Scientific, Rockford, IL). After blocking, the microarray sandwich was washed in Tris-buffered saline (TBS-buffer pH 7.4; 0.2 M NaCl, 5.5 mM KCl, and 0.02 M Tris), and serum was placed between the two slides and incubated for 2 h. To remove excess antibody, the microarray sandwich was washed as described above followed by the addition of secondary antibody (Alexa Fluor 647-conjugated Goat Anti-Bovine IgG (H + L); Jackson ImmunoResearch Laboratories Inc., West Grove, PA, USA). The slide was washed, centrifuged to dry, and scanned using a Microarray scanner (GenePix 4000B, Molecular Devices, Sunnyvale, CA, USA). Positive and negative controls were obtained from BLV-infected and BLV-uninfected cattle, respectively. Fluorescent signals were acquired with a microarray scanner at a resolution with a pixel size of 10 μm. Signals obtained were normalized and plotted to reflect the relative intensities of the fluorescence signals.

**Table 3:** The comparison results of four cattle samples for a peptide microarray

No.	Peptide sequence	Cattle^b^ no.				No.	Peptide sequence	Cattle^b^ no.				No.	Peptide sequence	Cattle^b^ no.			
		JB						JB						JB			
		S4	S6	N1	N2			S4	S6	N1	N2			S4	S6	N1	N2
p15^a^						p24^a^						gp51^a^					
1	MGNSPSYNPPAGISP	−	+	+	−	28	DLRSQYQNLWLQAWK	−	+	−	−	12	RYTLDSVNGYPKIYW	−	−	−	+
2	PSYNPPAGISPSDWL	−	−	+	−	29	QYQNLWLQAWKNLPT	−	−	−	−	13	DSVNGYPKIYWPPPQ	−	−	−	−
3	PPAGISPSDWLNLVQ	−	−	−	−	30	LWLQAWKNLPTRPSV	−	−	−	−	14	GYPKIYWPPPQGRRR	−	+	−	−
4	ISPSDWLNLVQSAQR	+	−	−	−	31	AWKNLPTRPSVQPWS	−	−	−	−	15	IYWPPPQGRRRFGAR	+	+	+	−
5	DWLNLVQSAQRLNPR	−	−	−	+	32	LPTRPSVQPWSTIVQ	+	−	−	−	*16*	*PPQGRRRFGARAMVT*	*+*	*+*	*+*	*+*
6	LVQSAQRLNPRPSPS	−	−	−	−	33	PSVQPWSTIVQGPAE	−	−	+	−	17	RRRFGARAMVTYDCE	−	+	+	+
7	AQRLNPRPSPSDFTD	−	−	−	−	34	PWSTIVQGPAESYVE	−	−	−	−	18	GARAMVTYDCEPRCP	+	−	−	−
8	NPRPSPSDFTDLKNY	−	−	−	−	35	IVQGPAESYVEFVNR	−	−	−	−	19	MVTYDCEPRCPYVGA	−	−	−	−
9	SPSDFTDLKNYIHWF	−	−	−	+	36	PAESYVEFVNRLQIS	−	+	−	−	20	DCEPRCPYVGADRFD	−	−	−	−
10	FTDLKNYIHWFHKTQ	−	−	−	+	37	YVEFVNRLQISLADN	−	−	−	−	21	RCPYVGADRFDCPHW	−	−	−	−
11	KNYIHWFHKTQKKPW	−	−	−	−	38	VNRLQISLADNLPDG	−	+	−	−	22	VGADRFDCPHWDNAS	−	−	−	−
12	HWFHKTQKKPWTFTS	−	−	−	−	39	QISLADNLPDGVPKE	−	+	+	+	23	RFDCPHWDNASQADQ	−	−	−	−
13	KTQKKPWTFTSGGPT	−	−	−	−	40	ADNLPDGVPKEPIID	−	−	+	−	24	PHWDNASQADQGSFY	−	−	−	−
14	KPWTFTSGGPTSCPP	−	−	−	−	41	PDGVPKEPIIDSLSY	−	−	−	−	25	NASQADQGSFYVNHQ	−	−	−	−
15	FTSGGPTSCPPGRFG	−	−	−	−	42	PKEPIIDSLSYANAN	−	−	+	−	26	ADQGSFYVNHQILFL	−	−	−	−
16	GPTSCPPGRFGRVPL	−	−	−	−	43	IIDSLSYANANKECQ	−	−	+	−	27	SFYVNHQILFLHLKQ	−	−	−	−
17	CPPGRFGRVPLVLAT	−	−	−	−	44	LSYANANKECQQILQ	−	−	+	−	28	NHQILFLHLKQCHGI	−	−	−	−
18	RFGRVPLVLATLNEV	−	−	−	+	45	NANKECQQILQGRGL	−	−	+	−	29	LFLHLKQCHGIFTLT	−	−	−	−
19	VPLVLATLNEVLSNE	−	−	−	−	46	ECQQILQGRGLVAAP	−	−	−	−	30	LKQCHGIFTLTWEIW	−	−	+	−
20	LATLNEVLSNEGGAP	−	+	+	−	47	ILQGRGLVAAPVGQK	−	+	−	−	31	HGIFTLTWEIWGYDP	−	+	+	−
21	NEVLSNEGGAPGASA	−	−	−	−	48	RGLVAAPVGQKLQAC	−	−	−	−	32	TLTWEIWGYDPLITF	−	−	+	+
22	SNEGGAPGASAPEEQ	−	−	−	−	49	AAPVGQKLQACAHWA	−	−	−	−	33	EIWGYDPLITFSLHK	−	−	−	−
23	GAPGASAPEEQPPPY	−	−	+	−	50	GQKLQACAHWAPKMK	−	−	−	−	34	YDPLITFSLHKIPDP	−	−	+	−
24	ASAPEEQPPPYDPPA	−	−	−	−	51	QACAHWAPKMKQPAI	−	−	−	−	35	ITFSLHKIPDPPQPD	−	+	−	−
25	EEQPPPYDPPAI	−	−	+	−	52	HWAPKMKQPAIL	−	−	−	−	36	LHKIPDPPQPDFPQL	−	−	−	−
p24^a^						p12^a^						37	PDPPQPDFPQLNSDW	−	−	+	−
												38	QPDFPQLNSDWVPSV	−	−	−	−
1	LPIISEGNRNRHRAW	+	−	+	−	1	VHTPGPKMPGPRQPA	+	+	+	−	39	PQLNSDWVPSVRSWA	−	−	−	−
2	SEGNRNRHRAWALRE	+	−	+	−	2	GPKMPGPRQPAPKRP	−	−	−	−	40	SDWVPSVRSWALLLN	−	+	+	−
3	RNRHRAWALRELQDI	+	+	+	−	3	PGPRQPAPKRPPPGP	−	−	−	−	41	PSVRSWALLLNQTAR	−	−	−	−
4	RAWALRELQDIKKEI	+	+	+	−	4	QPAPKRPPPGPCYRC	−	−	−	−	42	SWALLLNQTARAFPD	−	−	+	−
5	LRELQDIKKEIENKA	+	+	+	−	5	KRPPPGPCYRCLKEG	−	−	−	−	43	LLNQTARAFPDCAIC	−	−	−	−
6	QDIKKEIENKAPGSQ	+	−	+	−	6	PGPCYRCLKEGHWAR	−	−	−	−	44	TARAFPDCAICWEPS	−	−	−	−
7	KEIENKAPGSQVWIQ	+	−	+	−	7	YRCLKEGHWARDCPT	−	−	−	−	45	FPDCAICWEPSPPWA	−	−	−	−
8	NKAPGSQVWIQTLRL	−	−	+	−	8	KEGHWARDCPTKATG	−	+	+	+	46	AICWEPSPPWAPEIL	−	−	−	−
9	GSQVWIQTLRLAILQ	−	−	−	−	9	WARDCPTKATGPPPG	−	+	+	+	47	EPSPPWAPEILVYNK	−	−	−	−
10	WIQTLRLAILQADPT	−	−	−	−	10	CPTKATGPPPGPCPI	+	−	+	−	48	PWAPEILVYNKTISS	−	−	−	−
11	LRLAILQADPTPADL	+	−	−	−	11	ATGPPPGPCPICKDP	−	−	−	−	49	EILVYNKTISSSGPG	−	−	−	−
12	ILQADPTPADLEQLC	+	−	−	−	12	PPGPCPICKDPSHWK	+	+	+	−	50	YNKTISSSGPGLALP	−	−	−	−
13	DPTPADLEQLCQYIA	+	−	−	−	13	CPICKDPSHWKRDCP	−	−	−	−	51	ISSSGPGLALPDAQI	−	−	−	−
14	ADLEQLCQYIASPVD	−	−	+	−	14	KDPSHWKRDCPTLKS	−	+	+	−	52	GPGLALPDAQIFWVN	−	+	−	+
15	QLCQYIASPVDQTAH	+	−	+	−	gp51^a^						53	ALPDAQIFWVNTSSF	−	−	−	−
16	YIASPVDQTAHMTSL	−	−	+	−							54	AQIFWVNTSSFNTTQ	−	−	+	−
17	PVDQTAHMTSLTAAI	−	−	−	−	1	TWRCSLSLGNQQWMT	−	−	−	−	55	WVNTSSFNTTQGWHH	−	−	−	−
18	TAHMTSLTAAIAAAE	−	−	−	−	2	SLSLGNQQWMTAYNQ	−	−	−	−	56	SSFNTTQGWHHPSQR	−	−	+	−
19	TSLTAAIAAAEAANT	+	−	+	−	3	GNQQWMTAYNQEAKF	−	−	−	−	57	TTQGWHHPSQRLLFN	−	−	+	−
20	AAIAAAEAANTLQGF	−	−	−	−	4	WMTAYNQEAKFSISI	−	−	+	−	58	WHHPSQRLLFNVSQG	−	−	−	−
21	AAEAANTLQGFNPQN	−	−	+	−	5	YNQEAKFSISIDQIL	−	−	−	−	59	SQRLLFNVSQGNALL	−	−	−	−
22	ANTLQGFNPQNGTLT	−	−	−	+	6	AKFSISIDQILEAHN	−	−	+	−	60	LFNVSQGNALLLPPI	−	−	+	−
23	QGFNPQNGTLTQQSA	−	−	+	−	7	ISIDQILEAHNQSPF	−	−	+	−	61	SQGNALLLPPISLVN	−	−	+	−
24	PQNGTLTQQSAQPNA	−	−	+	−	8	QILEAHNQSPFCAKS	+	−	−	−	62	ALLLPPISLVNLSTA	−	−	+	−
25	TLTQQSAQPNAGDLR	−	−	+	−	9	AHNQSPFCAKSPRYT	−	−	−	−	63	PPISLVNLSTASSAP	−	−	−	−
26	QSAQPNAGDLRSQYQ	−	−	+	−	10	SPFCAKSPRYTLDSV	−	−	−	−	64	LVNLSTASSAPPTRV	−	−	+	−
27	PNAGDLRSQYQNLWL	−	+	−	−	11	AKSPRYTLDSVNGYP	−	−	−	−	65	STASSAPPTRVRR	−	−	−	−

Italic indicates a common B cell epitope which was identified in four BLV experimentally infected Japanese Black cattle by peptide microarray

+ only positive response with BLV positive serum; − none response with serums

^a^Antigens were derived from BLV Gag (p15, p24, and p12) and Env gp51 proteins

^b^These BLV positive serums were obtained from BLV-experimentally infected cattle as shown in Table 1

### Synthetic peptide containing a C-terminal cysteine, and a new high-throughput peptide ELISA system

A C-terminal cysteine-containing peptide (gp51p16-C) that contained the B cell epitope gp51p16 and its 17 variants (Fig. 2a) was synthesized and purified using high-pressure liquid chromatography (HPLC) to >70 % purity (Scrum. Inc., Tokyo, Japan). A new high-throughput peptide ELISA system was designed using cysteine-containing peptides to allow crosslinking with maleimide-activated carrier protein and mariculture keyhole limpet hemocyanin (mcKLH) (Thermo Fisher Scientific) to fix the peptide on the microplate well surface [57]. Briefly, mcKLH was dispensed into a 96-well flat bottom microplate (Sumitomo Bakelite Co., Ltd. Tokyo, Japan) and incubated overnight at 4 °C. The microplate was extensively washed in phosphate buffered saline (PBS) containing 0.05 % Tween 20 (PBS-T). The C-terminal cysteine-containing peptide was dissolved in maleimide conjugation buffer [83 mM sodium phosphate buffer (pH 7.2) containing 0.1 M EDTA, 0.9 M NaCl, and 0.02 % sodium azide] and added to the wells of the microplate. After incubation, the microplate was washed and blocked with PBS-T containing 10 % horse serum (Gibco, Grand Island, NY, USA). To absorb non-specific antibodies prior to add into microplate, the 500-fold diluted serum sample was mixed with 200 μg/ml KLH (Sigma, St. Louis, MO, USA), 20 μg/ml mcKLH and 10 % horse serum at 37 °C at least 30 min. After washing, peroxidase-conjugated Affinipure goat anti-bovine IgG (H + L) (Jackson ImmunoResearch Laboratories Inc.) was added to each well, and the microplate was read at an optical density (OD) of 450 nm with a microplate reader (EnSight; PerkinElmer, Yokohama, Japan). The cut-off line value was determined in accordance to histogram analysis using BLV negative and positive serum samples. All tests were set up in triplicate. The measured OD of the samples was compared with that of control wells incubated without peptide. The stimulation index (SI) was calculated using the following formula:

$${\text{Stimulation index}}\; = \;\frac{{{\text{gp}}51{\text{p}}16 \text{-}{\text{C or variant peptides}}}}{\text{no peptide}}$$

### Cell culture and transfection

Human embryonic kidney 293T cells, 23CLN cells, and FLK-BLV cells that had been productively infected with BLV [42] were cultured in Dulbecco’s modified Eagle’s medium (DMEM, Gibco) supplemented with penicillin, streptomycin, and glutamine (PSG, Gibco), and 10 % fetal bovine serum (FBS, Sigma). 293T cells (3 × 10^5^ cells) and 23CLN cells (5 × 10^5^ cells) were transfected with 3.6 and 7.6 μg of pBLV-IF [58], an infectious molecular clone of BLV with two copies of the long terminal repeat (LTR) and full-length BLV provirus, mutated pBLV-IFs, respectively, or a negative control vector [pBluescript II (KS^-^)] (Stratagene, La Jolla, CA), and 0.4 μg of pEGFP-N1 [59] using 12 μl of FuGENE HD (Promega, Madison, WI, USA) for 293T cells or 32 μl for 23CLN cells.

### Construction of mutant pBLV-IF

Alanine mutations were introduced into the *env* gene of pBLV-IF by site-directed mutagenesis. The 3′-half of the pBLV-IF sequence was cloned into a pBluescript II (SK^-^) vector using *Hind*III and *Kpn*I restriction enzyme sites, and the plasmid was subjected to site-directed mutagenesis by PCR using PrimeSTAR GXL (Takara Bio, Otsu, Japan) as follows: 98 °C for 2 min, followed by 25–30 cycles of 98 °C for 15 s, and 68 °C for 90 s. The primer pairs used to generate the alanine mutations are described in Table 4. Each 3′-half of pBLV-IF encoding the alanine mutation in the *env* sequence was digested with the restriction enzymes *Hind*III and *Bmg*BI, and each DNA fragment was cloned into the corresponding region of pBLV-IF in full-length.

**Table 4:** Oligonucleotide primers for generation of pBLV-IF mutants

Mutant	Forward primer sequence	Reverse primer sequence
R7A	gctCGCCGGTTTGGAGCCAG	agcCCCTTGTGGGGGGGGCCAGT
R9A	CGCgctTTTGGAGCCAGGGCCA	AAAagcGCGCCGCCCTTGTGG
F10A	CGCCGGgctGGAGCCAGGGCCA	agcCCGGCGCCGCCCTTGTGG
V16A	GCCATGgctACATATGATTGCGAGCC	ATATGTagcCATGGCCCTGGCTCCAAACC
Y18A	GTCACAgctGATTGCGAGCCCCGATGC	GCAATCagcTGTGACCATGGCCCTGGC

The sequences corresponding to alanine substitution are underlined

### Syncytium formation assay

Thirty-six hours following transfection, 293T cells were cultured and syncytium formation was determined. The cells were visualized using the confocal laser scanning microscope FV1000 (Olympus, Tokyo, Japan).

### Flow cytometer and Western blotting

A fraction of the 23CLN cells were harvested 20 h post-transfection, and the ratio of GFP-expressing cells was determined using a FACSCalibur™ flow cytometer (BD Japan, Tokyo, Japan). Data were analyzed using FCS Express (version 3; De Novo Software, Los Angeles, CA, USA). The remaining 23CLN cells were lysed 48 h following transfection, and lysates with equal numbers of GFP-expressing cells were subjected to Western blotting with an anti-BLV Env protein monoclonal antibody (Mab) (BLV-2; VMRD, Pullman, WA, USA) and an anti-BLV Gag protein Mab (BLV-3; VMRD, Pullman, WA, USA) followed by incubation with HRP-conjugated goat anti-mouse IgG (Amersham Bioscience, Piscataway, NJ) as previously described [60].

### Analysis of sequences

Amino acid sequences were downloaded from the GenBank database and aligned using the MEGA software version 6.06 [61].

## References

[1] Aida Y, Murakami H, Takahashi M, Takeshima SN (2013). Mechanisms of pathogenesis induced by bovine leukemia virus as a model for human T-cell leukemia virus. Front Microbiol.

[2] Burny A, Cleuter Y, Kettmann R, Mammerickx M, Marbaix G, Portetelle D, van den Broeke A, Willems L, Thomas R (1988). Bovine leukaemia: facts and hypotheses derived from the study of an infectious cancer. Vet Microbiol.

[3] Gillet N, Florins A, Boxus M, Burteau C, Nigro A, Vandermeers F, Balon H, Bouzar AB, Defoiche J, Burny A (2007). Mechanisms of leukemogenesis induced by bovine leukemia virus: prospects for novel anti-retroviral therapies in human. Retrovirology.

[4] Ott SL, Johnson R, Wells SJ (2003). Association between bovine-leukosis virus seroprevalence and herd-level productivity on US dairy farms. Prev Vet Med.

[5] Erskine RJ, Bartlett PC, Byrem TM, Render CL, Febvay C, Houseman JT (2012). Association between bovine leukemia virus, production, and population age in Michigan dairy herds. J Dairy Sci.

[6] Bartlett PC, Norby B, Byrem TM, Parmelee A, Ledergerber JT, Erskine RJ (2013). Bovine leukemia virus and cow longevity in Michigan dairy herds. J Dairy Sci.

[7] Rhodes JK, Pelzer KD, Johnson YJ, Russek-Cohen E (2003). Comparison of culling rates among dairy cows grouped on the basis of serologic status for bovine leukemia virus. J Am Vet Med Assoc.

[8] Sagata N, Yasunaga T, Ohishi K, Tsuzuku-Kawamura J, Onuma M, Ikawa Y (1984). Comparison of the entire genomes of bovine leukemia virus and human T-cell leukemia virus and characterization of their unidentified open reading frames. EMBO J.

[9] Sagata N, Yasunaga T, Ogawa Y, Tsuzuku-Kawamura J, Ikawa Y (1984). Bovine leukemia virus: unique structural features of its long terminal repeats and its evolutionary relationship to human T-cell leukemia virus. Proc Natl Acad Sci USA.

[10] Dube S, Bachman S, Spicer T, Love J, Choi D, Esteban E, Ferrer JF, Poiesz BJ (1997). Degenerate and specific PCR assays for the detection of bovine leukaemia virus and primate T cell leukaemia/lymphoma virus pol DNA and RNA: phylogenetic comparisons of amplified sequences from cattle and primates from around the world. J Gen Virol.

[11] Hamard-Peron E, Muriaux D (2011). Retroviral matrix and lipids, the intimate interaction. Retrovirology.

[12] Copeland TD, Morgan MA, Oroszlan S (1983). Complete amino acid sequence of the nucleic acid-binding protein of bovine leukemia virus. FEBS Lett.

[13] Mager A, Masengo R, Mammerickx M, Letesson JJ (1994). T cell proliferative response to bovine leukaemia virus (BLV): identification of T cell epitopes on the major core protein (p24) in BLV-infected cattle with normal haematological values. J Gen Virol.

[14] Willems L, Kerkhofs P, Attenelle L, Burny A, Portetelle D, Kettmann R (1997). The major homology region of bovine leukaemia virus p24gag is required for virus infectivity in vivo. J Gen Virol.

[15] Katoh I, Yasunaga T, Yoshinaka Y (1993). Bovine leukemia virus RNA sequences involved in dimerization and specific gag protein binding: close relation to the packaging sites of avian, murine, and human retroviruses. J Virol.

[16] Portetelle D, Couez D, Bruck C, Kettmann R, Mammerickx M, Van der Maaten M, Brasseur R, Burny A (1989). Antigenic variants of bovine leukemia virus (BLV) are defined by amino acid substitutions in the NH_2_ part of the envelope glycoprotein gp51. Virology.

[17] Callebaut I, Voneche V, Mager A, Fumiere O, Krchnak V, Merza M, Zavada J, Mammerickx M, Burny A, Portetelle D (1993). Mapping of B-neutralizing and T-helper cell epitopes on the bovine leukemia virus external glycoprotein gp51. J Virol.

[18] Voneche V, Portetelle D, Kettmann R, Willems L, Limbach K, Paoletti E, Ruysschaert JM, Burny A, Brasseur R (1992). Fusogenic segments of bovine leukemia virus and simian immunodeficiency virus are interchangeable and mediate fusion by means of oblique insertion in the lipid bilayer of their target cells. Proc Natl Acad Sci USA.

[19] Hill AV (1998). The immunogenetics of human infectious diseases. Annu Rev Immunol.

[20] Aida Y, Takeshima SN, Baldwin CL, Kaushik AK, Dorian JG, Anatoly R (2015). Bovine immunogenetics. The Genetics of cattle.

[21] Lewin HA, Russell GC, Glass EJ (1999). Comparative organization and function of the major histocompatibility complex of domesticated cattle. Immunol Rev.

[22] Juliarena MA, Poli M, Sala L, Ceriani C, Gutierrez S, Dolcini G, Rodriguez EM, Marino B, Rodriguez-Dubra C, Esteban EN (2008). Association of BLV infection profiles with alleles of the BoLA-DRB3.2 gene. Anim Genet.

[23] Mirsky ML, Olmstead C, Da Y, Lewin HA (1998). Reduced bovine leukaemia virus proviral load in genetically resistant cattle. Anim Genet.

[24] Miyasaka T, Takeshima SN, Jimba M, Matsumoto Y, Kobayashi N, Matsuhashi T, Sentsui H, Aida Y (2013). Identification of bovine leukocyte antigen class II haplotypes associated with variations in bovine leukemia virus proviral load in Japanese Black cattle. Tissue Antigens.

[25] Bai L, Takeshima SN, Isogai E, Kohara J, Aida Y (2015). Novel CD8 cytotoxic T cell epitopes in bovine leukemia virus with cattle. Vaccine.

[26] Sakakibara N, Kabeya H, Ohashi K, Sugimoto C, Onuma M (1998). Epitope mapping of bovine leukemia virus transactivator protein Tax. J Vet Med Sci.

[27] Kabeya H, Ohashi K, Sugimoto C, Onuma M (1999). Bovine leukaemia virus envelope peptides cause immunomodulation in BALB/c mice. Vet Immunol Immunopathol.

[28] Gatei MH, Good MF, Daniel RC, Lavin MF (1993). T-cell responses to highly conserved CD4 and CD8 epitopes on the outer membrane protein of bovine leukemia virus: relevance to vaccine development. J Virol.

[29] Portetelle D, Dandoy C, Burny A, Zavada J, Siakkou H, Gras-Masse H, Drobecq H, Tartar A (1989). Synthetic peptides approach to identification of epitopes on bovine leukemia virus envelope glycoprotein gp51. Virology.

[30] Radke K, Grossman D, Kidd LC (1990). Humoral immune response of experimentally infected sheep defines two early periods of bovine leukemia virus replication. Microb Pathog.

[31] Onuma M, Hodatsu T, Yamamoto S, Higashihara M, Masu S, Mikami T, Izawa H (1984). Protection by vaccination against bovine leukemia virus infection in sheep. Am J Vet Res.

[32] Suneya M, Onuma M, Yamamoto S, Hamada K, Watarai S, Mikami T, Izawa H (1984). Induction of lymphosarcoma in sheep inoculated with bovine leukaemia virus. J Comp Pathol.

[33] Onuma M, Wada M, Yasutomi Y, Yamamoto M, Okada HM, Kawakami Y (1990). Suppression of immunological responses in rabbits experimentally infected with bovine leukemia virus. Vet Microbiol.

[34] Davydov II, Tonevitskii AG (2009). Linear B-cell epitope prediction. Mol Biol (Mosk).

[35] Eichmann K, Rajewsky K (1975). Induction of T and B cell immunity by anti-idiotypic antibody. Eur J Immunol.

[36] Reineke U, Schutkowski M (2009). Epitope mapping protocols. Preface. Methods Mol Biol.

[37] Inabe K, Ikuta K, Aida Y (1998). Transmission and propagation in cell culture of virus produced by cells transfected with an infectious molecular clone of bovine leukemia virus. Virology.

[38] Tajima S, Takahashi M, Takeshima SN, Konnai S, Yin SA, Watarai S, Tanaka Y, Onuma M, Okada K, Aida Y (2003). A mutant form of the tax protein of bovine leukemia virus (BLV), with enhanced transactivation activity, increases expression and propagation of BLV in vitro but not in vivo. J Virol.

[39] Zhu H, Luo H, Yan M, Zuo X, Li QZ (2015). Autoantigen microarray for high-throughput autoantibody profiling in systemic lupus erythematosus. Genom Proteom Bioinform.

[40] Yakobson B, Brenner J, Ungar-Waron H, Trainin Z (1998). Short-termed expression of interleukin-12 during experimental BLV infection may direct disease progression to persistent lymphocytosis. Vet Immunol Immunopathol.

[41] Rovnak J, Boyd AL, Casey JW, Gonda MA, Jensen WA, Cockerell GL (1993). Pathogenicity of molecularly cloned bovine leukemia virus. J Virol.

[42] Van Der Maaten MJ, Miller JM (1975). Replication of bovine leukemia virus in monolayer cell cultures. Bibl Haematol.

[43] Gutierrez G, Rodriguez SM, de Brogniez A, Gillet N, Golime R, Burny A, Jaworski JP, Alvarez I, Vagnoni L, Trono K, Willems L (2014). Vaccination against delta-retroviruses: the bovine leukemia virus paradigm. Viruses.

[44] Rodriguez SM, Florins A, Gillet N, de Brogniez A, Sanchez-Alcaraz MT, Boxus M, Boulanger F, Gutierrez G, Trono K, Alvarez I (2011). Preventive and therapeutic strategies for bovine leukemia virus: lessons for HTLV. Viruses.

[45] Palacios R, Martinez-Maza O, Guy K (1983). Monoclonal antibodies against HLA-DR antigens replace T helper cells in activation of B lymphocytes. Proc Natl Acad Sci USA.

[46] Mourad W, Scholl P, Diaz A, Geha R, Chatila T (1989). The staphylococcal toxic shock syndrome toxin 1 triggers B cell proliferation and differentiation via major histocompatibility complex-unrestricted cognate T/B cell interaction. J Exp Med.

[47] Palker TJ, Riggs ER, Spragion DE, Muir AJ, Scearce RM, Randall RR, McAdams MW, McKnight A, Clapham PR, Weiss RA (1992). Mapping of homologous, amino-terminal neutralizing regions of human T-cell lymphotropic virus type I and II gp46 envelope glycoproteins. J Virol.

[48] Callebaut I, Portetelle D, Burny A, Mornon JP (1994). Identification of functional sites on bovine leukemia virus envelope glycoproteins using structural and immunological data. Eur J Biochem.

[49] Johnston ER, Albritton LM, Radke K (2002). Envelope proteins containing single amino acid substitutions support a structural model of the receptor-binding domain of bovine leukemia virus surface protein. J Virol.

[50] de Brogniez A, Bouzar AB, Jacques JR, Cosse JP, Gillet N, Callebaut I, Reichert M, Willems L (2015). Mutation of a single envelope N-Linked glycosylation site enhances the pathogenicity of bovine leukemia virus. J Virol.

[51] Jimba M, Takeshima SN, Matoba K, Endoh D, Aida Y (2010). BLV-CoCoMo-qPCR: quantitation of bovine leukemia virus proviral load using the CoCoMo algorithm. Retrovirology.

[52] Jimba M, Takeshima SN, Murakami H, Kohara J, Kobayashi N, Matsuhashi T, Ohmori T, Nunoya T, Aida Y (2012). BLV-CoCoMo-qPCR: a useful tool for evaluating bovine leukemia virus infection status. BMC Vet Res.

[53] Takeshima SN, Kitamura-Muramatsu Y, Yuan Y, Polat M, Saito S, Aida Y (2015). BLV-CoCoMo-qPCR-2: improvements to the BLV-CoCoMo-qPCR assay for bovine leukemia virus by reducing primer degeneracy and constructing an optimal standard curve. Arch Virol.

[54] Polat M, Ohno A, Takeshima SN, Kim J, Kikuya M, Matsumoto Y, Mingala CN, Onuma M, Aida Y (2015). Detection and molecular characterization of bovine leukemia virus in Philippine cattle. Arch Virol.

[55] Takeshima SN, Matsumoto Y, Miyasaka T, Arainga-Ramirez M, Saito H, Onuma M, Aida Y (2011). A new method for typing bovine major histocompatibility complex class II DRB3 alleles by combining two established PCR sequence-based techniques. Tissue Antigens.

[56] Lavinder JJ, Wine Y, Giesecke C, Ippolito GC, Horton AP, Lungu OI, Hoi KH, DeKosky BJ, Murrin EM, Wirth MM (2014). Identification and characterization of the constituent human serum antibodies elicited by vaccination. Proc Natl Acad Sci USA.

[57] Cuccuru MA, Dessi D, Rappelli P, Fiori PL (2012). A simple, rapid and inexpensive technique to bind small peptides to polystyrene surfaces for immunoenzymatic assays. J Immunol Methods.

[58] Inabe K, Nishizawa M, Tajima S, Ikuta K, Aida Y (1999). The YXXL sequences of a transmembrane protein of bovine leukemia virus are required for viral entry and incorporation of viral envelope protein into virions. J Virol.

[59] Cormack BP, Valdivia RH, Falkow S (1996). FACS-optimized mutants of the green fluorescent protein (GFP). Gene.

[60] Murakami T, Aida Y (2014). Visualizing Vpr-induced G2 arrest and apoptosis. PLoS One.

[61] Tamura K, Peterson D, Peterson N, Stecher G, Nei M, Kumar S (2011). MEGA5: molecular evolutionary genetics analysis using maximum likelihood, evolutionary distance, and maximum parsimony methods. Mol Biol Evol.

